# Therapeutic potential of PIMSR, a novel CB1 receptor neutral antagonist, for cocaine use disorder: evidence from preclinical research

**DOI:** 10.1038/s41398-022-02059-w

**Published:** 2022-07-18

**Authors:** Ewa Galaj, Briana Hempel, Allamar Moore, Benjamin Klein, Guo-Hua Bi, Eliot L. Gardner, Herbert H. Seltzman, Zheng-Xiong Xi

**Affiliations:** 1 Addiction Biology Unit, Molecular Targets and Medication Discovery Branch, Intramural Research Program, National Institute on Drug Abuse, Baltimore, MD USA; 2 Department of Psychological and Brain Sciences, Colgate University, Hamilton, NY Canada; 3 Neuropsychopharmacology Section, Molecular Targets and Medication Discovery Branch, Intramural Research Program, National Institute on Drug Abuse, Baltimore, MD USA; 4 Center for Drug Discovery, Research Triangle Institute, Research Triangle Park, NC USA

**Keywords:** Pharmacodynamics, Molecular neuroscience

## Abstract

Cannabinoid CB1 receptors (CB1Rs) have been major targets in medication development for the treatment of substance use disorders. However, clinical trials with rimonabant, a CB1R antagonist/inverse agonist, failed due to severe side effects. Here, we evaluated the therapeutic potential of PIMSR, a neutral CB1R antagonist lacking an inverse agonist profile, against cocaine’s behavioral effects in experimental animals. We found that systemic administration of PIMSR dose-dependently inhibited cocaine self-administration under fixed-ratio (FR5), but not FR1, reinforcement, shifted the cocaine self-administration dose-response curve downward, decreased incentive motivation to seek cocaine under progressive-ratio reinforcement, and reduced cue-induced reinstatement of cocaine seeking. PIMSR also inhibited oral sucrose self-administration. Importantly, PIMSR alone is neither rewarding nor aversive as assessed by place conditioning. We then used intracranial self-stimulation (ICSS) to explore the possible involvement of the mesolimbic dopamine system in PIMSR’s action. We found that PIMSR dose-dependently attenuated cocaine-enhanced ICSS maintained by electrical stimulation of the medial forebrain bundle in rats. PIMSR itself failed to alter electrical ICSS, but dose-dependently inhibited ICSS maintained by optical stimulation of midbrain dopamine neurons in transgenic DAT-Cre mice, suggesting the involvement of dopamine-dependent mechanisms. Lastly, we examined the CB1R mechanisms underlying PIMSR’s action. We found that PIMSR pretreatment attenuated Δ^9^-tetrahydrocannabinol (Δ^9^-THC)- or ACEA (a selective CB1R agonist)-induced reduction in optical ICSS. Together, our findings suggest that the neutral CB1R antagonist PIMSR deserves further research as a promising pharmacotherapeutic for cocaine use disorder.

## Introduction

Cocaine use disorder (CUD) is characterized by continued drug use despite clinically significant distress and other negative consequences. Currently, there is no available pharmacological treatment that has proven effective in replicated, randomized, placebo-controlled clinical trials. To remedy this, medication discovery research has prioritized the search for effective CUD medications. Over the past decade, CB1 receptors (CB1Rs) have been given much attention because of their abundant presence in the central nervous system (CNS) [1–4] and critical involvement in addiction [5–7]. Preclinical studies have demonstrated that mice with genetic ablation of CB1Rs can acquire drug self-administration after extensive training but generally tend to self-administer lower amounts of psychostimulants and are less motivated to seek the drug than their control littermates [6–8]. In contrast, cannabinoid receptor agonists such as Δ^9^-THC, CP55,940 or WIN55,212-2 have been shown to excite midbrain dopamine (DA) neurons [9], produce conditioned place preference (CPP) [10], enhance electrical brain stimulation reward [11, 12] and trigger reinstatement of extinguished drug seeking [13] under certain experimental conditions, which has been generally believed to be mediated mainly by activation of CB1Rs [5, 11–13].

Given the importance of CB1Rs in drug-related behavior, CB1R antagonists have been proposed as potential therapeutic candidates for the treatment of drug abuse and addiction [14–17]. A number of preclinical studies with rimonabant and AM251, selective CB1R antagonists with inverse agonist profiles, suggested that they reduce self-administration of heroin [18–20], methamphetamine [21, 22], alcohol [23–26], nicotine [27], and to some extent cocaine[7, 28]; but see [29–31]. In addition, pharmacological blockade of CB1Rs can significantly attenuate the development and expression of cocaine-induced CPP [32, 33] and prevent relapse to addictive drug use (including nicotine, alcohol, and psychostimulants [34–42]), making CB1R ligands promising candidates for the treatment of CUD.

Despite CB1R antagonists being effective in reducing drug reward and relapse in experimental animals (for comprehensive reviews, see [5, 43]), CB1R antagonists have significant adverse side effects including nausea, emesis, depression, and suicidal tendencies in humans [15, 44–48]. As a consequence, clinical trials with CB1R antagonists were abruptly terminated and the US Food and Drug Administration (FDA) decided not to approve CB1R ligands until better safety and efficacy data become available.

Recently, the medication discovery community has shifted research interest to CB1R neutral antagonists presuming that the inverse agonist profile of CB1R antagonists may underlie their unwanted side effects. We and others recently reported that CB1R neutral antagonists such as AM4113 offer attractive prospects for pharmacotherapeutic exploration as they do not produce psychotropic side effects [49–52], nor do they produce aversive or rewarding effects on their own [19]. Here we evaluated the therapeutic potential of PIMSR, a novel CB1R neutral antagonist, for the treatment of CUD, using a series of animal models of drug abuse and addiction. In vitro radioligand binding assays indicate that PIMSR has as high affinity (Ki = 17–57 nM) for human CB1Rs expressed in cultured HEK cells as does rimonabant (Ki = 1.8–18 nM) [53]. Computational molecular modeling (CB1R docking) and Ca^++^ channel assays indicate that PIMSR is able to block WIN55,212-2-induced inhibition of Ca^++^ influx, while itself failing to alter Ca^++^ influx [53]. PIMSR also stabilizes both the active and inactive states of CB1Rs, revealing the molecular interactions that mechanistically confer the property of neutral antagonism [53]. Electrophysiological assays indicate that co-administration of PIMSR can reverse the inhibitory effects of Δ^9^-THC or synthetic cannabinoids (AM2201, AM018) on excitatory glutamate transmission in the hippocampus [54]. Pharmacodynamic assays indicate that PIMSR displays relatively low brain penetration — reflected by a brain: plasma concentration ratio of 0.24 after intraperitoneal (i.p.) administration of 10 mg/kg [55]. Importantly, systemic administration of PIMSR significantly reduces body weight, food intake, and adiposity as well as improving glycemic control and lipid homeostasis in high-fat diet-induced obese mice, but also shows increased alanine transaminase (ALT) and liver weight as markers of liver injury with chronic administration [55]. This is unlikely due to neutral CB1R antagonism itself since other neutral antagonists (AM6545) do not produce liver injury or other unwanted side effects [56]. In contrast, repeated (3-day) administration of PIMSR significantly reduced high-dose alcohol-induced acute hepatic injury and steatosis in C57BL/6J mice [55], suggesting the additional potential utility of PIMSR in the treatment of obesity and alcohol use disorder.

Therefore, in the present study, we first systemically evaluated PIMSR’s pharmacological efficacy in reducing cocaine-taking and cocaine-seeking behavior in experimental animals, and then explored the underlying neural mechanisms using electrophysiological, optogenetic, and transgenic approaches. We found that PIMSR reduces cocaine reward and relapse by inhibition of the mesolimbic DA system via both CB1R and non-CB1R mechanisms.

## Materials and methods

### Animals

Subjects consisted of 48 male Long-Evans rats (purchased from Charles River Laboratories, Frederick, MD), 42 male and female wildtype (C57/BL6J) mice, and 15 heterozygous DAT-cre mice (breeders purchased from Jackson Laboratory, Bar Harbor, ME; B6.SJL-*Slc6a3*^*tm1.1(Cre)Bkmn*^/J; stock # 006660), aged 4–24 weeks. Rats and mice were chosen based on the availability of the test drug (PIMSR), transgenic animals (DAT-cre), and equipment systems (rat electrical ICSS *vs*. mouse optical ICSS) in the laboratory. To the best of our knowledge, there are no reports of sex differences in animal behavioral responses to PIMSR. Animals were housed in climate-controlled animal colony rooms on a reversed light-dark cycle (lights on at 7:00 p.m., lights off at 7:00 a.m.) with free access to food and water throughout the study. The housing conditions and animal care were consistent with the *Guide for the Care and Use of Laboratory Animals* (National Research Council, 2011). All experimental procedures were approved by the National Institute on Drug Abuse Animal Care and Use Committee.

#### Exp. 1: Cocaine self-administration and cue-induced reinstatement of drug seeking in rats

Intravenous catheterization surgery and cocaine self-administration procedures were performed as described previously [19, 28]. After stable cocaine self-administration was achieved, the effects of PIMSR (3, 10, 30 mg/kg, i.p.) or vehicle (equal injection volume of 5% Kolliphor EL) on cocaine self-administration under fixed-ratio (FR1, FR5), progressive-ratio (PR), and multiple cocaine dose conditions were evaluated. In addition, the effects of PIMSR on cue-induced reinstatement were also evaluated in separate groups of rats (see more details in the S.I.).

#### Exp. 2: Oral sucrose self-administration in mice

Procedures for oral sucrose self-administration in mice were the same as we reported previously [57]. This experiment was designed to determine whether the same doses of PIMSR inhibit non-drug reinforced behavior (see experimental details in the S.I.).

#### Exp. 3: PIMSR-induced place conditioning in mice

This experiment was designed to determine whether PIMSR itself is rewarding or aversive. The experimental details are provided in the S.I.

#### Exp. 4: Electrical intracranial self-stimulation (ICSS) in rats

To determine the neural mechanisms underlying PIMSR’s action, we observed the effects of PIMSR on rewarding intracranial self-stimulation (ICSS) behavior in the presence or absence of cocaine. The experimental procedures are the same as we reported previously [12] (also see the S.I. for more details).

#### Exp. 5: Optogenetic intracranial self-stimulation (ICSS) in DAT-Cre mice

To determine whether DA-dependent and CB1 receptor-dependent mechanisms underlie the effects of PIMSR on cocaine self-administration and brain-stimulation reward, we measured the effects of PIMSR on Δ^9^-THC- or ACEA-induced changes in ICSS maintained by optical activation of ventral tegmental area (VTA) DA neurons in DAT-Cre mice expressing Cre-recombinase under the DA transporter (DAT) promoter. The optical ICSS procedures are the same as we reported previously [58] (see details in the S.I.)

#### Exp. 6: Δ^9^-THC-induced tetrad effects in mice

Lastly, we examined the ability of PIMSR to antagonize high dose Δ^9^-THC-induced classical cannabinoid tetrad effects—analgesia, hypothermia, catalepsy and immobility in mice. The procedures for the measurement of Δ^9^-THC-induced tetrad effects are the same as we reported previously [59] (see the S.I. for details).

### Data analysis

All values were presented as means ± SEM. Animal group sizes were chosen based on a power analysis (*n* = 6–12 per group) and extensive previous experience with the animal models used. No data points were excluded from the analysis in any experiment. The investigators were blinded to the group allocation during the experiments and when assessing the outcome. Where a variation in group size occurred, this was due to animals being dropped from the experiment due to obstruction or clogging of i.v. catheters.

To validate the use of parametric statistics, we performed a Shapiro Wilk Test for data normality evaluation and Levene’s test for homogeneity for between-subject ANOVA. The group size (*n* > 5) is the number of independent values (individual animals), and statistical analysis was done using these independent values. One-way or two-way repeated-measures analysis of variance (RM ANOVA) was used to evaluate the effects of PIMSR on cocaine or sucrose self-administration, cue-induced reinstatement, or cannabinoid-induced changes in oICSS. The post-hoc group comparisons were conducted only if the ANOVA F value achieved *p* < 0.05. Estimation statistics were used when necessary (when data were not normally distributed (www.estimationstats.com). The value of *p* < 0.05 was used to indicate statistically significant differences among or between groups.

## Results

### PIMSR fails to alter cocaine self-administration under FR1 reinforcement

**Fig. 1:**
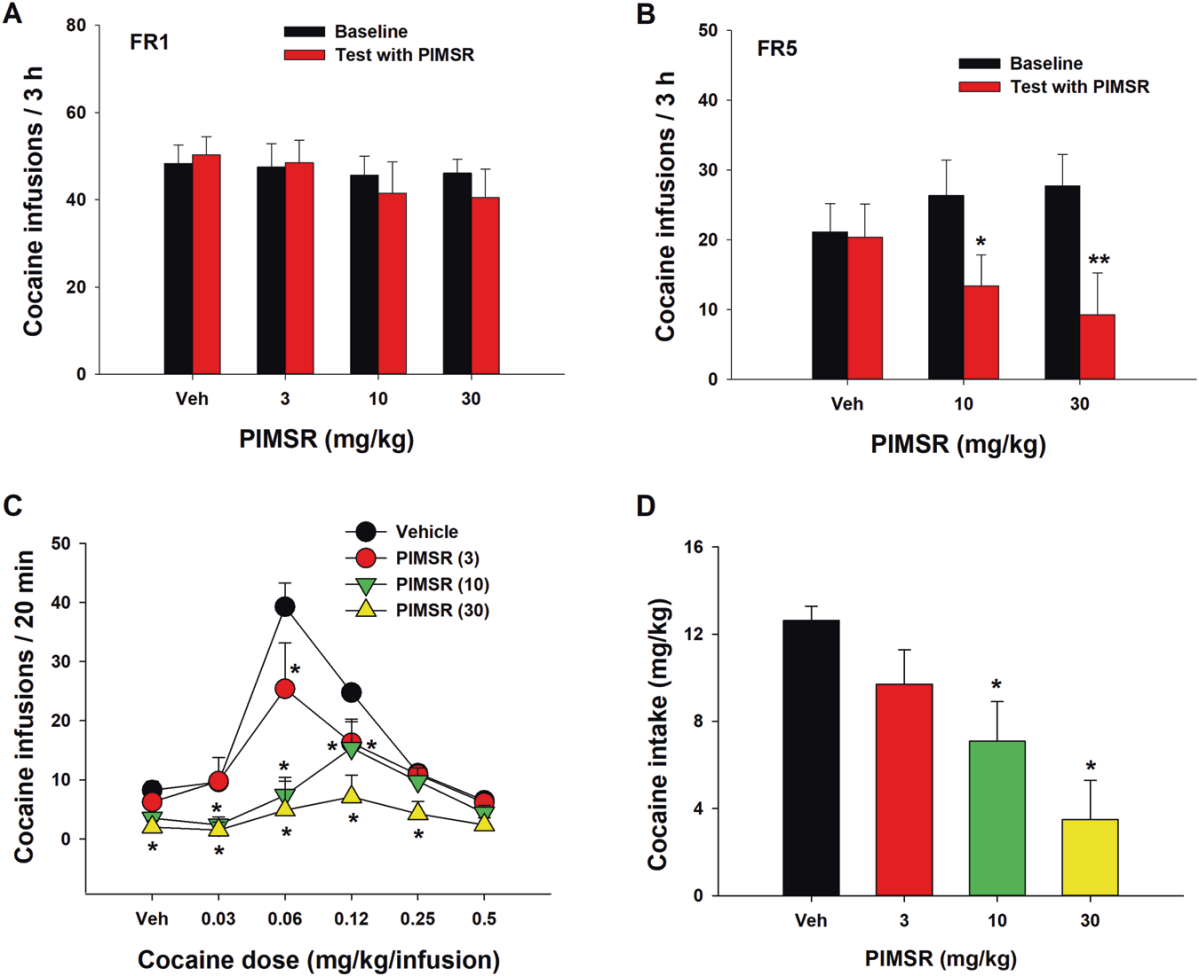
The effects of PIMSR on cocaine self-administration in rats under different schedules of reinforcement. **A**, **B** PIMSR failed to alter cocaine self-administration under FR1 reinforcement (*n* = 8), but dose-dependently inhibited cocaine self-administration under FR5 reinforcement in a separate group of rats (*n* = 8). **C** PIMSR significantly inhibited cocaine self-administration maintained by low-dose cocaine (0.03, 0.06, 0.12, 0.25 mg/kg/infusion) under FR2 reinforcement and shifted the cocaine dose-response curve downward (*n* = 8). **D** PIMSR dose-dependently reduced cocaine intake calculated from the data in **C** (i.e., sum of cocaine dose × infusions at each cocaine dose). *p < 0.05, **p < 0.01, com*p*ared to vehicle.

We first examined the effects of PIMSR on cocaine self-administration maintained by cocaine (0.5 mg/kg/infusion) under FR1 reinforcement. Figure 1A shows the total numbers of cocaine infusions earned within 3 h session under an FR1 reinforcement schedule, indicating that PIMSR, at 3, 10, and 30 mg/kg, failed to alter cocaine self-administration. A two-way RM ANOVA over PIMSR doses revealed no significant main effects of PIMSR dose (*F*_3,28_ = 0.54; *p* = 0.6) and no PIMSR dose × phase (baseline vs. Test with PIMSR) interaction (*F*_3,28_ = 2.17; *p* = 0.11). Similarly, no significant difference was observed in the number of active or inactive lever presses before and after each dose of PIMSR (data not shown), suggesting that PIMSR is unable to inhibit cocaine self-administration when high dose cocaine is available under easy access (FR1) conditions.

Next, we examined the effects of PIMSR on cocaine self-administration maintained by the same dose of cocaine (0.5 mg/kg/infusion) but under a FR5 reinforcement schedule, in which the animals needed to work harder to receive a cocaine reward. Figure 1B shows that the rats took less cocaine under FR5 than under the FR1 reinforcement (averaged cocaine infusions decreased from ~50 to ~20 within a 3-h session). PIMSR, at 10 and 30 mg/kg, significantly inhibited cocaine self-administration. A two-way RM ANOVA revealed a significant PIMSR treatment main effect (*F*_1, 7_ = 35.88, *p* < 0.001). Post-hoc individual group comparisons revealed a significant reduction in the total number of cocaine infusions after 10 mg/kg (*p* < 0.05) or 30 mg/kg (*p* < 0.01) PIMSR compared to either vehicle or baseline. These findings suggest that PIMSR was effective in attenuating cocaine self-administration when a high dose of cocaine is unavailable without effort or the cumulative dose of cocaine is not high.

### PIMSR reduces low-dose cocaine self-administration under FR2 reinforcement

We then assessed the pharmacological efficacy of PIMSR on a full dose range (0.5, 0.25, 0.125, 0.06, 0.03 mg/kg/infusion) of cocaine self-administration under a higher FR (FR2) requirement. We observed a typical inverted U-shaped dose-response curve for cocaine self-administration after vehicle treatment (Fig. 1C). Systemic treatment with PIMSR (3, 10, or 30 mg/kg) dose-dependently decreased cocaine self-administration maintained by lower doses (<0.5 mg/kg/infusion) of cocaine under FR2 reinforcement and shifted the cocaine dose-response curve downward. A two-way ANOVA revealed a significant cocaine dose × PIMSR treatment interaction (*F*_15,140_ = 3.19, *p* < 0.05). Post-hoc Tukey tests for multiple group comparisons revealed that the 30 mg/kg dose of PIMSR significantly reduced self-administration of cocaine doses from 0.03 to 0.25 mg/kg/infusion, while the 10 mg/kg dose of PIMSR significantly reduced self-administration of cocaine doses from 0.03 to 0.12 mg/kg/infusion, and the 3 mg/kg dose of PIMSR significantly reduced self-administration of cocaine doses from 0.06 to 0.12 mg/kg/infusion. PIMSR caused dose-dependent reductions in overall cocaine intake (i.e., sum of cocaine intake under the different doses of cocaine, mg/kg/session), as shown in Fig. 1D. A one-way ANOVA revealed a significant PIMSR treatment main effect (*F*_3,28_ = 6.4, *p* < 0.01) with 10 and 30 mg/kg being the effective doses.

### PIMSR reduces motivation for cocaine as determined by self-administration by PR reinforcement

**Fig. 2:**
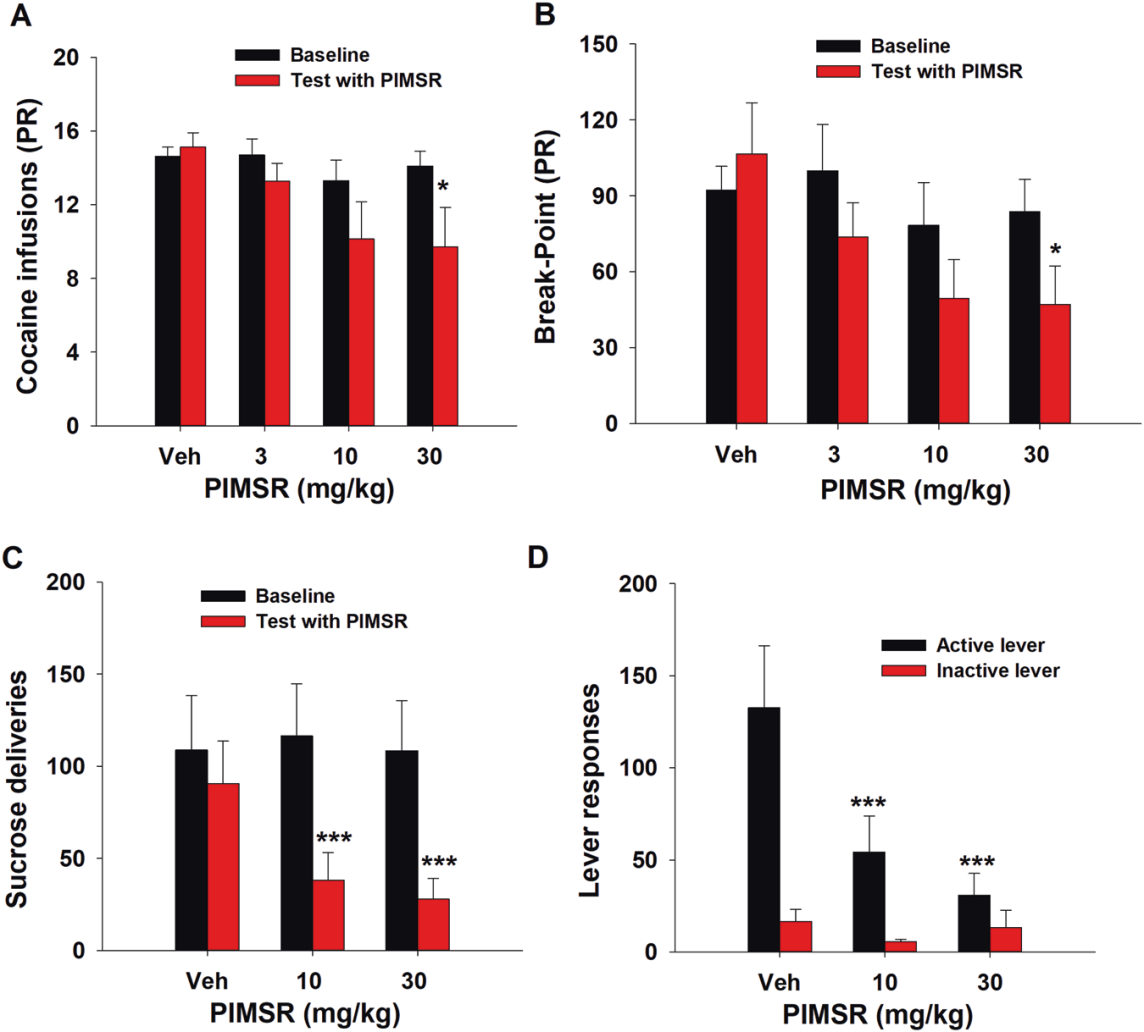
The effect of PIMSR on PR cocaine self-administration and FR1 oral sucrose self-administration. **A**, **B** PIMSR inhibited cocaine self-administration under PR reinforcement in a dose-dependent manner (*n* = 7), as assessed by the number of cocaine infusions (**A**) and break-point (**B**). **C**, **D** PIMSR dose-dependently inhibited oral sucrose self-administration under FR1 reinforcement (*n* = 6), as assessed by the number of sucrose deliveries (**C**) and active lever responses (**D**). PIMSR did not alter inactive lever responses. *p < 0.05, ***p < 0.001, com*p*ared to vehicle.

To determine whether PIMSR reduces motivation to seek cocaine, we examined the effects of PIMSR on cocaine self-administration under PR reinforcement. Figure 2A shows that rats in all dose groups displayed similar baseline (before PIMSR treatment) cocaine infusions, while PIMSR produced a dose-dependent reduction in the number of cocaine infusions under PR reinforcement. A two-way ANOVA revealed a significant PIMSR dose main effect (*F*_3,24_ = 2.88, *p* < 0.05). Post hoc individual group comparisons revealed a significant reduction in the number of cocaine infusions following 30 mg/kg PIMSR (*p* < 0.05, Fig. 2A). Similarly, PIMSR also produced a dose-dependent reduction in break point for cocaine-taking behavior. A two-way ANOVA revealed a significant PIMSR dose main effect (*F*_3,24_ = 3.04, *p* < 0.05). Post hoc individual group comparisons revealed a significant reduction in break point following 30 mg/kg PIMSR (*p* < 0.05, Fig. 2B), suggesting that PIMSR (at a sufficiently high dose) was effective in attenuating animals’ motivation for cocaine.

### PIMSR reduces oral sucrose self-administration

To determine whether the PIMSR actions observed above are drug- or cocaine-specific, we observed the effects of PIMSR on oral sucrose self-administration in mice. We chose mice in this experiment because of the limited drug source. We found that PIMSR, at 10 and 30 mg/kg, also significantly reduced sucrose self-administration (Fig. 2C). Two-way RM ANOVA revealed a significant PIMSR treatment main effect (*F*_1, 5_ = 21.32, *p* < 0.01). Post-hoc individual group comparisons revealed a significant reduction (*p* < 0.001) in sucrose deliveries after 10 or 30 mg/kg PIMSR when compared to the vehicle control group. Figure 2D shows the active and inactive lever responses for sucrose reward. Two-way ANOVA revealed a significant PIMSR treatment main effect (*F*_2, 10_ = 72.36, *p* < 0.001). Post hoc-individual group comparisons revealed a significant reduction (*p* < 0.001) in active lever responding after 10 mg/kg or 30 mg/kg PIMSR administration, while no difference was observed in inactive active responses.

### PIMSR reduces cue-induced reinstatement of cocaine seeking

**Fig. 3:**
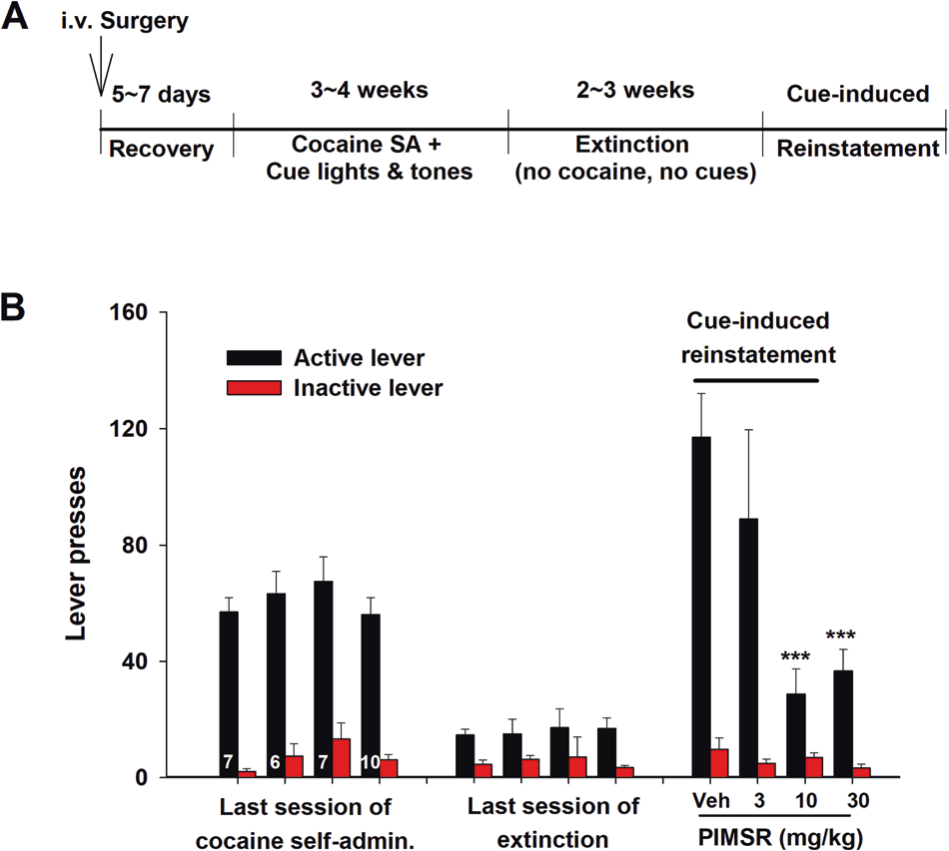
Effects of PIMSR on cue-induced reinstatement of cocaine seeking in rats. **A** General experimental procedures for cue-induced reinstatement of cocaine seeking; **B** The averaged active and inactive lever responses during the last three days of cocaine self-administration, the last three days of extinction, and reinstatement testing. PIMSR, when administered 30 min prior to reinstatement testing, dose-dependently reduced cue-induced reinstatement of cocaine-seeking behavior (*n* = 6–10 per group). ***p < 0.001, compared to vehicle.

We next examined the action of PIMSR on drug-paired environmental cue-induced reinstatement of drug-seeking behavior. Figure 3A, B shows the general experimental procedure and the averaged active *vs*. inactive lever responses during the last three sessions of cocaine self-administration, extinction, and reinstatement testing. Rats in all PIMSR dose groups showed similar levels of responding during the last days of self-administration and extinction. On the reinstatement test day, re-exposure to cocaine-associated cues (lights and tones) produced robust reinstatement responding on the active lever in the absence of PIMSR (0 mg/kg). However, PIMSR, at 10 and 30 mg/kg, produced a significant reduction in cue-induced active lever presses. A two-way ANOVA performed on cue-induced reinstatement responding revealed a significant PIMSR main effect (*F*_3,26_ = 5.08, *p* < 0.001), inactive vs. inactive lever response main effect (*F*_1,26_ = 48.59, *p* < 0.001), and a significant PIMSR × lever response interaction (*F*_3,26_ = 5.35, *p* < 0.01). Post-hoc individual group comparisons revealed a significant reduction in cue-induced active lever responding after 10 mg/kg or 30 mg/kg (*p* < 0.001), but not 3 mg/kg, PIMSR pretreatment. These findings suggest that PIMSR has therapeutic potential for relapse prevention.

### PIMSR is neither rewarding nor aversive in mice

**Fig. 4:**
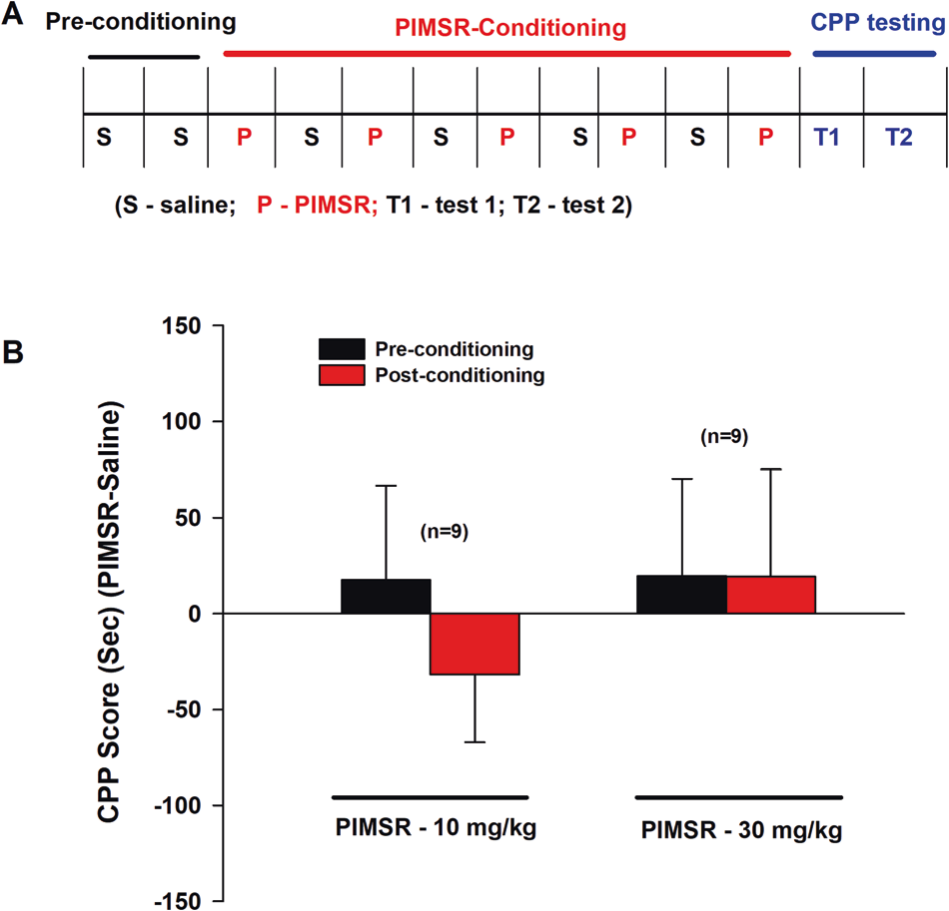
PIMSR-induced CPP or CPA in mice. **A** General CPP procedures. **B** The CPP score (i.e., the averaged value of the two tests) after 10 mg/kg or 30 mg/kg PIMSR-conditioning (*n* = 9), indicating that PIMSR is neither rewarding nor aversive.

We then used CPP to examine whether PIMSR itself has similar aversive or depressive effects as rimonabant. Figure 4A shows the general experimental procedures. PIMSR, at 10 or 30 mg/kg, did not produce rewarding or aversive effects as assessed by CPP (Fig. 4B). A two-way RM ANOVA did not reveal a significant conditioning main effect (*F*_1,16_ = 0.33, *p* > 0.05), PIMSR dose main effect (*F*_1,16_ = 0.28, *p* > 0.05) or conditioning × dose interaction (*F*_1,16_ = 0.33, *p* > 0.05), suggesting a lack of rewarding or aversive effects by PIMSR itself.

### PIMSR attenuates cocaine-enhanced rewarding electrical ICSS

**Fig. 5:**
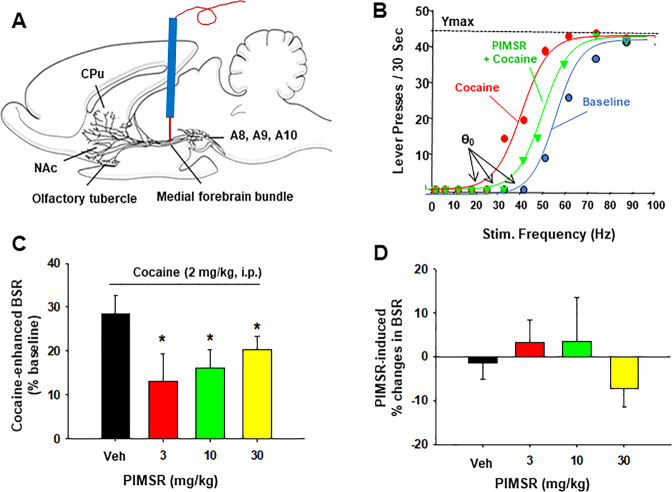
The effects of PIMSR on electrical ICSS in rats. **A** A schematic diagram showing the experimental methods. **B** Representative stimulation-response curves, illustrating the stimulation threshold (*θ*_*0*_), *Y*_max_, and the effects of cocaine and/or PIMSR on the stimulation-response curve. Cocaine (2 mg/kg, i.p.) shifted the stimulation-response curve to the left and decreased the stimulation threshold (*θ*_*0*_ value), which was ameliorated by PIMSR. **C** Cocaine-induced reduction in the *θ*_*0*_ value (% baseline) in the absence or presence of PIMSR, indicating that cocaine alone produced an enhancement in ICSS (as less stimulation current was required to initiate ICSS in the presence of cocaine). This effect was attenuated by pretreatment with PIMSR. **D** PIMSR alone (*n* = 12) did not alter the *θ*_*0*_ value, suggesting that it is not rewarding or aversive by itself. *p < 0.05, compared to vehicle.

To determine possible mechanisms underlying PIMSR’s action, we observed the interactions of cocaine and PIMSR on electrical ICSS. Figure 5A shows that electrical stimulation of the medial forebrain bundle (MFB) at the level of the hypothalamus produced robust ICSS behavior (lever-pressing to receive the stimulation). Figure 5B shows representative rate-frequency functions for ICSS, indicating ICSS threshold (θ_0_), *Y*_max_, and the effects of cocaine in the presence or absence of PIMSR. Cocaine (2 mg/kg, i.p.) significantly shifted the curve to the left and decreased the ICSS θ_0_ value without affecting asymptotic rates of responding (i.e., no change in *Y*_max_ level) (Fig. 5B), indicating that cocaine produced an enhancement (additive or synergistic effects) on ICSS. PIMSR (3, 10 or 30 mg/kg, i.p.) pretreatment significantly attenuated cocaine-enhanced ICSS as assessed by *θ*_0_ value (Fig. 5C). A one-way ANOVA with PIMSR dose as a within-subjects factor revealed a significant PIMSR treatment main effect (*F*_3,33_ = 3.79; *p* = 0.019). Post-hoc Tukey tests showed that PIMSR significantly attenuated cocaine-enhanced ICSS. Strikingly, PIMSR itself failed to alter the ICSS threshold θ_0_ value (Fig. 5D, *F*_3,33_ = 2.475, *p* = 0.101), suggesting that PIMSR is not rewarding or aversive by itself, which is consistent with the finding in the CPP experiment and its neutral CB1R antagonist profile.

### PIMSR reduces optical ICSS in DAT-Cre mice

**Fig. 6:**
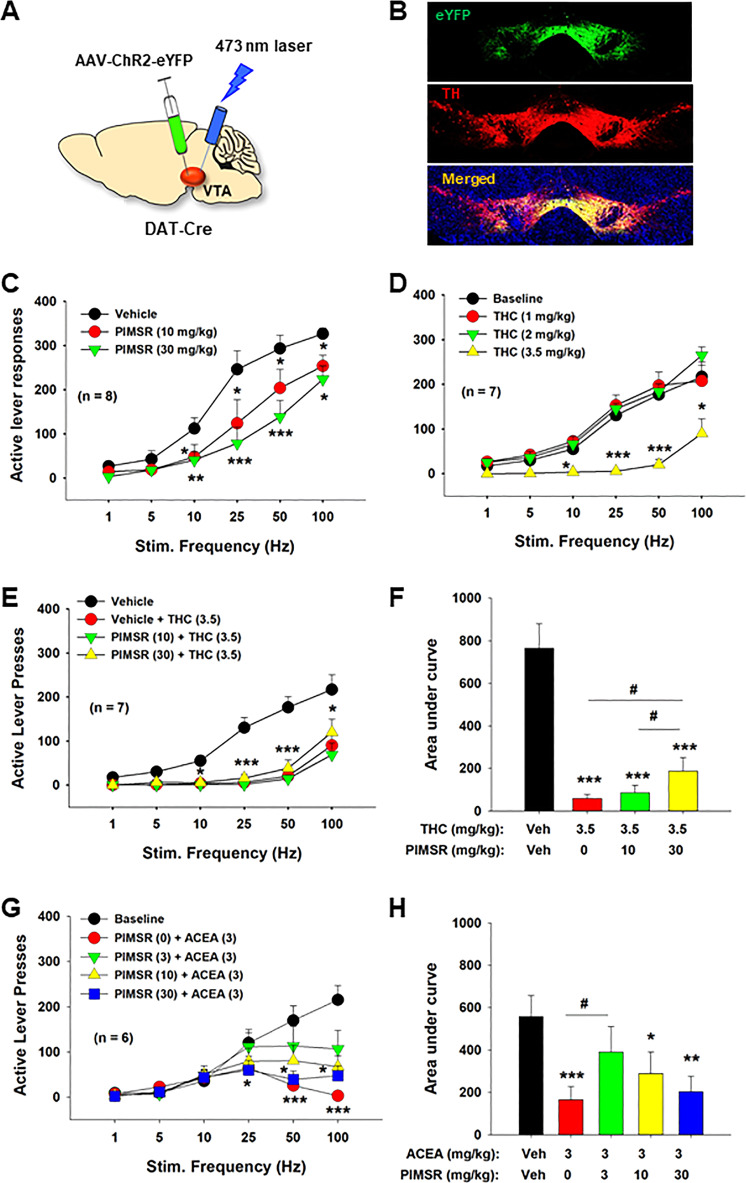
Effects of PIMSR on oICSS maintained by optical stimulation of VTA DA neurons in DAT-Cre mice. **A** A diagram showing the experimental methods for oICSS. **B** Representative images of TH-immunostaining (red) and fluorescent ChR2-eYFP expression (green) in the VTA, illustrating TH and ChR2-eYFP colocalization in VTA DA neurons. **C** Systemic administration of PIMSR inhibited optical ICSS (*n* = 8). **D** Δ^9^-THC alone also inhibited oICSS at a high dose (3.5 mg/kg) and shifted the stimulation-response curve downward (*n* = 7). **E** Pretreatment with PIMSR failed to significantly alter Δ^9^-THC-induced reduction in oICSS (*n* = 7). **F** However, the AUC assays for the data shown in **E** revealed a significant reduction in Δ^9^-THC’s action on oICSS after PIMSR pretreatment. **G** PIMSR also attenuated ACEA-induced reduction in oICSS (*n* = 6). **H** The AUC assays for the data shown in **G** revealed a significant reduction in ACEA action on oICSS after 3 mg/kg PIMSR pretreatment. *p < 0.05, compared to vehicle at each stimulation frequency. #*p* < 0.05, com*p*ared to Δ^9^-THC or ACEA treatment group.

We then examined whether a DA-dependent mechanism underlies PIMSR action against cocaine’s rewarding effects. Figure 6A illustrates the experimental set-up, demonstrating that AAV-ChR2-eYFP was injected into the VTA ipsilaterally, and optrodes (optical fibers) were surgically implanted 1 mm above the VTA in DAT-cre mice. Figure 6B shows fluorescent ChR2-eYFP and tyrosine hydroxylase (TH) colocalization confirming that ChR2 was expressed in VTA DA neurons. Unexpectedly, PIMSR (10, 30 mg/kg) alone caused significant reductions in optical ICSS maintained by stimulation of VTA DA neurons as shown in Fig. 6C. A two-way RM ANOVA with stimulation frequency and PIMSR dose as within-subjects factors revealed significant main effects of PIMSR dose (*F*_2,14_ = 12.73, *p* < 0.001), frequency (*F*_5,35_ = 92.47, *p* < 0.001 and a stimulation frequency × PIMSR interaction (*F*_10,70_ = 3.41, *p* < 0.001). Post-hoc Tukey tests for multiple group comparisons revealed that 10 or 30 mg/kg PIMSR were effective in attenuating oICSS responding (*p* < 0.05).

### PIMSR attenuates Δ^9^-THC-induced reduction of oICSS in DAT-cre mice

Next, we examined whether PIMSR is able to block the action produced by Δ^9^-THC using oICSS. We have previously demonstrated that Δ^9^-THC inhibits oICSS caused by optical stimulation of VTA DA neurons [60] in mice. In line with these findings, systemic administration of Δ^9^-THC, at 3.5 mg/kg, significantly inhibited oICSS in DAT-Cre mice (Fig. 6D). A two-way RM ANOVA revealed a significant Δ^9^-THC treatment main effect (*F*_3,18_ = 11.65, *p* < 0.001). Unexpectedly, pretreatment with PIMSR (30 min prior to Δ^9^-THC) failed to effectively block Δ^9^-THC-induced reduction in oICSS (Fig. 6F). A two-way RM ANOVA with frequency and treatment as within-subjects factors revealed significant main effects of treatment (Fig. 6E, *F*_3,18_ = 18.07, *p* < 0.001) and stimulation frequency (*F*_5,30_ = 33.27, *p* < 0.001) and a significant frequency × treatment interaction (*F*_15,90_ = 6.40, *p* < 0.001). Post-hoc Tukey tests for multiple group comparisons revealed attenuated responding in the groups administered 3.5 mg/kg Δ^9^-THC in the presence or absence of PIMSR (Fig. 6E).

Given that PIMSR displayed a trend toward reduction in Δ^9^-THC action in a dose-dependent manor, we then re-analyzed the data using the area under curve (AUC) for the data shown in Fig. 5E. We found that PIMSR pretreatment produced a significant reduction in Δ^9^-THC’s action on oICSS (Fig. 6F). One-way RM ANOVA revealed a significant PIMSR treatment main effect (*F*_3,18_ = 24.34, *p* < 0.001). Post-hoc individual group comparisons revealed a significant reduction in Δ^9^-THC action on oICSS after 30 mg/kg PIMSR pretreatment, suggesting that a CB1R mechanism at least in part underlies Δ^9^-THC-induced attenuation of oICSS.

### PIMSR attenuates ACEA-induced reduction of oICSS in DAT-cre mice

To further explore this finding, we examined the effects of PIMSR pretreatment on ACEA (a selective CB1R agonist)-induced reduction of oICSS. Figure 6G, H shows that ACEA, at 3 mg/kg, produced a significant reduction in oICSS, an effect similar to that which we reported previously [60]. Pretreatment with PIMSR, at 3 mg/kg, significantly blocked the action produced by ACEA, although this blockade effect is not PIMSR dose-dependent. A two-way RM ANOVA for the data shown in Fig. 6G revealed a significant PIMSR treatment main effect (*F*_4, 20_ = 3.21, *p* < 0.05), stimulation frequency main effect (*F*_5, 25_ = 8.68, *p* < 0.001), and treatment × frequency interaction (*F*_20, 100_ = 2.95, *p* < 0.001). One-way ANOVA for the AUC data shown in Fig. 6H also revealed a significant PIMSR treatment main effect (*F*_4,20_ = 7.83, *p* < 0.001). Post-hoc individual group comparisons revealed a significant reduction (*p* < 0.05) in ACEA action after 3 mg/kg, but not 10 mg/kg or 30 mg/kg, PIMSR administration.

### PIMSR fails to block Δ^9^-THC-induced tetrad effects in mice

Lastly, we examined whether PIMSR antagonizes the effects of Δ^9^-THC in the tetrad test. As shown in Fig. S1, Δ^9^-THC, at 10 mg/kg or 30 mg/kg (i.p.), produced characteristic cannabimimetic effects — hypothermia, analgesia, catalepsy, and immobility in wildtype mice. Systemic pretreatment with PIMSR (10 mg/kg, i.p.) failed to block Δ^9^-THC-induced analgesia, catalepsy, hypothermia, and immobility, suggesting that PIMSR, at the dose tested, doesn’t exert a clear CB1R antagonist behavioral profile. This finding suggests that higher doses of PIMSR may be required to block such high dose Δ^9^-THC-induced tetrad effects or other non-CB1R-mediated mechanisms may be involved in Δ^9^-THC’s action(s) in the tetrad test [59].

## Discussion

In the present study, we demonstrate that PIMSR failed to alter cocaine self-administration under a low cost (FR1) and high payoff (i.e., high dose cocaine − 0.5 mg/kg/infusion) reinforcement schedule, but dose-dependently inhibited cocaine self-administration under high cost (FR5) reinforcement or self-administration maintained by lower doses of cocaine (0.03, 0.06, 0.12, and 0.25 mg/kg/infusion) under a FR2 reinforcement schedule. PIMSR shifted the cocaine dose-response curve downward, and decreased cocaine intake. PIMSR also dose-dependently decreased break-point (a measure of incentive motivation) for cocaine taking under a PR schedule of reinforcement. Cue-induced reinstatement of cocaine-seeking was attenuated by PIMSR pretreatment, suggestive of an ability to reduce relapse. In addition, PIMSR also dose-dependently inhibited oral sucrose self-administration, suggesting that PIMSR action is not cocaine-specific. This is consistent with previous reports that CB1Rs are critically involved in the modulation of body weight, food intake, and energy metabolism [23, 44, 45, 47, 49, 50, 52, 55]. The observed reduction in cocaine (or sucrose) taking and seeking is unlikely caused by PIMSR-induced motoric incapacitation since PIMSR pretreatment failed to alter cocaine self-administration under a FR1 reinforcement schedule or reduce inactive lever responding under multiple experimental conditions. Neither did PIMSR affect *Y*_max_ values in electrical ICSS, a reliable indicator of locomotor incapacity. PIMSR alone also failed to produce locomotor impairment as assessed by catalepsy and rotarod locomotor performance. Importantly, PIMSR itself is neither rewarding nor aversive as assessed by CPP/CPA in mice, suggesting that PIMSR may not have abuse potential or produce unpleasant dysphoria in humans.

The above findings are supported by our electrical ICSS data demonstrating that systemic administration of PIMSR significantly reduced cocaine-enhanced ICSS. The anti-reward effects of PIMSR indicate that the mesolimbic DA system could be its target. Importantly, PIMSR by itself failed to alter electrical ICSS in rats and as such may not produce unwanted side effects such as depressed mood.

### CB1R antagonists and inverse agonists for cocaine use disorder

As mentioned above, CB1R full antagonists with inverse agonist profiles (such as SR141716A, AM251, and taranabant) were once thought to be promising candidates for the treatment of substance use disorders [5, 43, 61]. Indeed, SR141716A (commonly known as rimonabant) was shown to effectively reduce cocaine-induced hyperlocomotion [62], cocaine sensitization [30, 63], cocaine-induced CPP [64], and cue- or drug-induced, but not stress-induced reinstatement of cocaine seeking [30, 34, 39]. However, prior work examining the ability of other CB1R ligands to suppress cocaine self-administration has been inconsistent. For example, we have previously demonstrated that AM251 significantly lowered the break-point for cocaine self-administration under PR reinforcement in rats while rimonabant did not [28]. Neither AM251 nor SR141716 altered cocaine self-administration under a FR schedule of reinforcement [28–31, 61], suggesting that AM251 and SR141716A are not efficacious in attenuating cocaine self-administration under low cost (FR1, FR2) and high-payoff (cocaine doses from 0.5 to 1.0 mg/kg/infusion) experimental conditions. Furthermore, although mice with a genetic deletion of CB1Rs (CB1-KO) can acquire cocaine self-administration after extensive training [7, 8], they take less drug relative to wildtype mice [8, 65] and show a significant reduction in PR responding [7], suggesting an important role for CB1Rs in cocaine self-administration.

### CB1R neutral antagonists for cocaine use disorder

In contrast to CB1R antagonists/inverse agonists, neutral CB1R antagonists appear to be devoid of negative side effects and became a target of interest in medication development research for this reason. We recently demonstrated that AM4113, another CB1R neutral antagonist, inhibited self-administration of heroin, but not cocaine or methamphetamine, under FR2 reinforcement conditions in rats without producing aversion [19]. Others showed that AM4113 reduced alcohol consumption [16] and nicotine intake in rats [52]. In addition, AM4113 has been shown to effectively reduce nicotine [52] and cocaine seeking in rats and non-human primates [27]. During a substitution test, when the drug of abuse (nicotine, Δ^9^-THC or cocaine) was replaced by AM4113, monkeys failed to self-administer it [27], indicating low abuse liability.

Similar to the findings with AM4113, PIMSR also failed to alter cocaine self-administration maintained by a high dose of cocaine (0.5 mg/kg/infusion) under low effort (FR1) reinforcement conditions. However, when the work demand for cocaine was increased from FR1 to FR5 or the cocaine doses were decreased from 0.5 mg/kg/infusion to 0.25, 0.125, 0.06, or 0.03 mg/kg/infusion, PIMSR dose-dependently inhibited cocaine self-administration under FR and PR reinforcement schedules, suggesting that PIMSR is effective in reducing cocaine’s rewarding effects when high doses of cocaine are not freely available. In addition, PIMSR dose-dependently attenuated cue-induced reinstatement of drug-seeking, suggesting that this compound may be also efficacious in attenuating relapse to drug-seeking behavior.

### Rewarding *vs*. aversive effects of neutral CB1R antagonists

In the present report, some conflicting findings emerged between electrical ICSS *vs*. oICSS. Neither PIMSR nor AM4113 have any effect on ICSS in rats [19], which suggests that both neutral CB1R antagonists produce no affective valence on their own. In contrast, a high dose of SR141716A produces significant inhibition of ICSS [19], which aligns with clinical reports demonstrating mood-depressant side effects of SR141716A. However, in the oICSS assay PIMSR produced a mild, but significant, reduction in oICSS maintained by stimulation of VTA DA neurons in DAT-Cre mice, suggestive of an aversive or reward-attenuating effect. Caution is warranted in interpreting this finding for a number of reasons. First, PIMSR inhibition of oICSS may not necessarily equate to a diffuse negative effect on mood as this compound likely acts on CB1Rs expressed in multiple cell types. CB1R blockade on different neuronal phenotypes may have varied or opposite effects on brain reward function. For example, optical stimulation of DA neurons or glutamate neurons within the VTA is rewarding [60, 66, 67], while stimulation of VTA GABA neurons is aversive as assessed by oICSS and optical real-time place preference [58]. It is well known that VTA DA neurons receive both GABAergic and glutamatergic inputs [4, 68] and that CB1Rs are highly expressed on both GABA and glutamate neurons in the VTA and substantia nigra pars compacta (SNc) [66]. Thus, blockade of CB1Rs on both GABA and glutamate neurons by PIMSR would produce opposite effects in the mesolimbic DA system, and therefore, no-net change in DA-dependent behavior. This is supported by our finding that PIMSR is neither rewarding nor aversive in the CPP/CPA paradigm. This may also explain why PIMSR has no effect on electrical ICSS since electrical stimulation activates multiple phenotypes of neuronal fibers in the medial forebrain bundle, and PIMSR’s actions on different neural substrates may counteract each other. Second, PIMSR may not be a highly selective CB1R antagonist. We have recently reported that CB2Rs are highly expressed in midbrain DA neurons [5, 8]. Systemic administration of either CB2R agonists (BCP) or inverse agonists (Xie2-64) produces a significant reduction in oICSS by activation of CB2Rs in DA neurons [69, 70]. Thus, if PIMSR also has a binding affinity for CB2Rs, it would produce an inhibitory effect on oICSS. As such, the above finding implies that the mesolimbic DA system could be a particularly important and responsive target for PIMSR intervention.

### Receptor mechanisms underlying PIMSR effects

As stated above, computational modeling and in vitro receptor binding and functional assays indicate that PIMSR is a neutral CB1R antagonist [53]. Ex vivo electrophysiological studies have demonstrated that local bath perfusion of PIMSR is able to block cannabinoid (Δ^9^-THC, AM108, AM2201)-induced inhibition of presynaptic glutamate release or excitatory synaptic transmission in hippocampal brain slices [54]. In the present study, we found that pretreatment with PIMSR significantly attenuated Δ^9^-THC- or ACEA-induced reduction in oICSS, suggesting that CB1R blockade, at least in part, underlies cannabinoid action in oICSS and PIMSR-induced reduction in cocaine self-administration, cocaine-enhanced ICSS and cue-induced reinstatement of cocaine-seeking behavior observed in the present experiments.

We note that PIMSR failed to completely block Δ^9^-THC- or ACEA-induced reduction in oICSS nor did it alter high dose (10, 30 mg/kg) Δ^9^-THC-induced tetrad effects in mice. One possibility is that Δ^9^-THC is not a selective CB1R agonist. It also binds to other cannabinoid receptors such as CB2, GPR55, and PPARβ receptors [59, 69]. This may in part explain why PIMSR, a selective CB1R antagonist, cannot completely block Δ^9^-THC’s action. Another possibility is that in addition to binding to CB1Rs [53], PIMSR may also bind to other non-CB1 receptors at high doses, producing a reduction in DA-dependent oICSS that compromises the action produced by the blockade of CB1Rs. A third possibility is that PIMSR may be metabolized faster in rodents, and therefore, it may be difficult to maintain sufficient PIMSR levels to block CB1Rs in the brain. This is supported by tissue distribution assays demonstrating that following an acute i.p. dosing of 10 mg/kg, extremely high levels of PIMSR were found in the liver and in fat tissue, while a markedly lower level of PIMSR was found in the brain (brain:plasma concentration ratio of 0.24) [55]. Thus, more studies are needed to further assess PIMSR’s potential off targets and its pharmacokinetic profiles in other species such as rats, nonhuman primates—including oral bioavailability and brain penetration ability.

In summary, the present study demonstrates that the neutral CB1R antagonist PIMSR produces significant inhibitory effects on cocaine self-administration, cocaine-enhanced brain-stimulation reward, and cue-induced reinstatement of drug-seeking, suggesting that PIMSR could be a candidate for the treatment of cocaine use disorder. Given the poor outcome of CB1R antagonists/inverse agonists in clinical trials, a thorough investigation and validation of PIMSR’s efficacy and safety across preclinical and clinical trials are recommended.

## Supplementary information

Supplementary Materials

## Data Availability

All data are available within the article or its supplementary material.
