# Supplementary material for: Therapeutic potential of PIMSR, a novel CB1 receptor neutral antagonist, for cocaine use disorder: evidence from preclinical research
Source: Transl Psychiatry. 2022 Jul 18;12:286. doi: 10.1038/s41398-022-02059-w (PMC9293959; doi:10.1038/s41398-022-02059-w)
Supplement: Supplementary file 1 — Supplementary Materials [file 41398_2022_2059_MOESM1_ESM.docx]

Translational Psychiatry

**Therapeutic potential of PIMSR, a novel CB1 receptor neutral antagonist, for cocaine use disorder: evidence from preclinical research**

Ewa Galaj^1,2,#^, Briana Hempel^1,#^, Allamar Moore^3^, Ben Klein^1^, Guo-Hua Bi^1^, Eliot Gardner^3^, Herbert H. Seltzman^4^ & Zheng-Xiong Xi^1,*^

**Supplementary Information**

**Materials and Methods**

**Exp. 1: Cocaine self-administration in rats**

***Surgery***

Rats used in cocaine self-administration experiments were implanted intravenously (i.v.) with a microrenathane catheter (Braintree Scientific Inc., Braintree, MA, USA). Each rat was anaesthetized first with xylazine/ketamine (10/90 mg/kg, i.p.) and a small incision made to the right of the midline of the neck to expose the external jugular vein. One end of the i.v. catheter was inserted into the vein with the catheter tip reaching the right atrium. The catheter was then secured to the vein with silk suture and the other end fed subcutaneously around the back of the neck to exit near the back of the skull, connected to a bent 24-gauge stainless steel cannula (Plastics One Inc., Roanoke, VA, USA). The catheter and the guide cannula were secured to the skull with four stainless steel screws threaded into the skull and dental cement. The incision was then sutured.

***Procedure***

Cocaine self-administration sessions were conducted in standard operant conditioning chambers (Med-Associates, Saint Albans, VT, USA), each housed in a sound-attenuating box. Each chamber was equipped with two retractable levers, a white light above the active lever and a drug line connected to a Razel syringe pump (Razel Scientific Instruments, Saint Albans, VT, USA) set at 3.33 rpm. Rats were trained to lever press for cocaine (0.5 mg/kg) under a FR1 schedule of reinforcement during daily 3-hour sessions. Responding on the active lever resulted in i.v. delivery of cocaine and exposure to a drug-paired white cue light and sound (tone) that lasted for the duration of the infusion. Responses on the inactive lever were recorded but had no consequences. To prevent drug overdose, each animal was limited to a maximum of 50 infusions per self-administration session. Once animals demonstrated a pattern of stable responding, defined as < 20% variability in daily cocaine intake across three consecutive sessions, and an active/inactive lever press ratio exceeding 2:1, they were assigned to the following 4 experiments to determine the effects of PIMSR on cocaine-taking and cocaine-seeking behavior under different experimental conditions.

*Effects of PIMSR on single high dose cocaine self-administration under FR1 or FR5 reinforcement*

After stable rates of responding were established, one group of rats (n=8) randomly received one of three doses of PIMSR (3, 10, 30 mg/kg, i.p.) or vehicle (equal injection volume of 5% Kolliphor EL, formerly known as Cremophor EL; Sigma Aldrich, St. Louis, MO, USA) 30 min prior to the test session under FR1 reinforcement. Another group of rats (n=8) randomly received vehicle, 10 mg/kg, or 30 mg/kg PIMSR 30 min prior to the test session under FR5 reinforcement. Animals then received an additional 5–7 days of self-administration of cocaine alone until a baseline response rate was reestablished prior to being tested with another dose of PIMSR. The order of testing with different doses of PIMSR was counterbalanced.

*Effects of PIMSR on multiple-dose cocaine self-administration*

To further explore PIMSR’s pharmacological efficacy, we assessed whether PIMSR reduces cocaine self-administration maintained by a full range of cocaine doses. Within each session, rats self-administered multiple cocaine doses (0, 0.031, 0.0625, 0.125, 0.25, and 0.5 mg/kg/infusion) every 20 min in a descending dose sequence under a FR2 schedule of reinforcement. Cocaine concentration was adjusted by changes in the infusion volumes and duration of pump activation. After stable cocaine self-administration was achieved as defined above, animals (n=8) received a systemic administration of PIMSR (0, 3, 10, 30 mg/kg, i.p.) 30 min prior to the test session and later were allowed to self-administer the different doses of cocaine under the same conditions.  Animals were tested with different doses of PIMSR once their self-administration baselines were re-established. Tests were conducted 3-5 days apart. The order of testing with different doses of PIMSR was counterbalanced.

*Effects of PIMSR on progressive-ratio cocaine self-administration*

Next, we evaluated whether PIMSR reduces cocaine reward and motivation for cocaine taking/seeking using a progressive-ratio (PR) reinforcement paradigm in a third group of rats.  Subjects were initially trained to self-administer cocaine under FR1 reinforcement, as outlined above. Rats with stable responding were placed on a PR schedule of reinforcement that required each animal to emit progressively greater numbers of responses (1, 2, 4, 6, 9, 12, 15, 20…) in order to obtain successive cocaine infusions during a session [1, 2].  Under this schedule, the requirement for lever pressing became so high that eventually the animals stopped responding [termed the “break-point” (BP)], which was defined as the maximum number of active lever presses completed for the last cocaine infusion prior to a 1-h period during which no infusions were obtained.  After a stable BP was achieved for 3 consecutive sessions, rats were then randomly divided into 3 groups to test the effects of 3 different doses of PIMSR (0, 3, 10 or 30 mg/kg, i.p.) on PR cocaine self-administration. On the test day rats were injected i.p. with PIMSR 30 minutes prior to the self-administration session. Since it is relatively difficult to re-establish a stable BP after each drug test, we used a between-subjects design rather than a within-subjects design to determine the dose-response effects of PIMSR on BP for cocaine (n=8 per group).

*Effect of PIMSR on cue-induced reinstatement of cocaine seeking*

In this experiment we assessed whether PIMSR reduces cue-induced reinstatement of cocaine-seeking behavior. Here three different groups of rats (n=8 in each group) were trained to self-administer cocaine (0.5 mg/kg/inf) under a FR1 schedule of reinforcement, as described in Exp. 1. After 2 weeks of self-administration, rats underwent extinction training during which responding on either lever produced no consequences. Once rats reached extinction criteria (< 20 lever presses within their 3-hour session on 3 consecutive days), they were randomly divided into 3 dose group to test the effects of PIMSR on cue-induced reinstatement of cocaine seeking. Rats were injected with one of the PIMSR doses (0, 3 or 30 mg), 30 minutes prior to the onset of the session, and two presentations of light/tone/pump cues were made 2 min apart at the beginning of the session in order to reinstate responding.  Each response on the active lever was reinforced with cocaine-associated cue lights and tones, but not with cocaine.  Responding on the inactive lever was not reinforced.

**Exp. 2: Oral sucrose self-administration in mice**

Procedures for oral sucrose self-administration in mice were the same as we reported previously [3]. Briefly, mice were trained to self-administer sucrose under an FR1 schedule of reinforcement during daily 3-hour sessions. Responding on the active lever activated the syringe pump causing the delivery of 5% liquid sucrose onto a liquid food receptacle (0.02 mL per delivery) and the presentation of the light/tone cue above the active lever. Responses on the inactive lever were counted but had no consequences. During the 4.2-second infusion period, additional responses on the active lever were recorded but did not lead to additional infusions. Animals were tested with different doses of PIMSR once stable sucrose self-administration was achieved, defined as (i) at least 20 sucrose rewards earned per 3-hour session, (ii) less than 20% variability in daily sucrose intake across two consecutive sessions, and (iii) an active/inactive lever press ratio exceeding 2:1. All animals (n=6) met these criteria before being tested with PIMSR.

**Exp. 3: PIMSR-induced place conditioning in mice**

Twenty-six male wildtype mice were purchased from Charles River Laboratories (Raleigh, NC) and housed in the animal facility at NIDA IRP under a reversed 12 h light–dark cycle (light on at 7:00 PM) with free access to food and water. All procedures were in accordance with the Guide for the Care and Use of Laboratory Animals, 8th edition, 2011).

The place conditioning apparatus (Med Associates, St Albans, VT) consisted of two side compartments (21 × 28 cm^2^) and a central gray connecting area (21 × 12.5 cm^2^); a sliding door separated each compartment from the connecting area. The two side compartments differed in wall color (black vs white) and floor type (net vs grid). The animals were divided randomly into 3 treatment groups. Each mouse was given two 15 min preconditioning sessions to explore the entire CPP apparatus. Animals displaying significant bias toward one compartment (defined by a time difference between the two compartments ≥200 s) during preconditioning were excluded from the study. CPP procedures consisted of 5 days of PIMSR (0, 10, 30 mg/kg, i.p.) conditioning in one compartment and 5 days of saline conditioning in another compartment in an alternating two day cycle (e.g., … PIMSR–saline–PIMSR–saline …). The compartment (white vs black) paired with each PIMSR dose was randomly selected. On conditioning days, each animal received one dose of PIMSR (0, 10, 30 mg/kg, i.p.) 30 min prior to confinement in the assigned compartment for 30 min. 24 h and 48 h after the final saline injection, animals were placed in the same three-chamber CPP apparatus for 15 min and the time spent in each compartment was recorded. The CPP score was calculated by the time difference (seconds) that an animal spent in the PIMSR-paired compartment *versus* the saline-paired compartment.

**Exp. 4: Electrical intracranial self-stimulation (ICSS) in rats**

To explore the neural mechanisms underlying PIMSR’s action, we observed the effects of PIMSR on ICSS in the presence or absence of cocaine. The experimental procedures were the same as we reported previously [4].

***Surgery***

Under 65 mg/kg [sodium pentobarbital](http://www.guidetopharmacology.org/GRAC/LigandDisplayForward?ligandId=5480) anesthesia, rats were surgically implanted with a unilateral monopolar stainless steel stimulating electrode (Plastics One, Roanoke, VA, USA) targeted at the medial forebrain bundle at the level of the lateral hypothalamus (stereotaxic coordinates from bregma: AP + 2.5 mm, ML + 1.7 mm, and DV − 8.4 mm) (Paxinos and Watson *The Rat Brain in Stereotaxic Coordinates*, 5^th^ Edition, Academic Press, San Diego, CA, 2004). A wire wrapped around a jeweler's screw implanted in the skull and connected to a mini-pin in the electrical connector at the top of the electrode was used to accommodate return electrical current. The electrodes were cemented to the skull with acrylic resin cement. Each animal was kept warm and under observation until all effects of the anesthetic had dissipated. Rats were monitored closely and allowed a minimum of 7 days to recover, prior to the start of experiments [12].

***Procedures***

Operant chambers for electrical ICSS were equipped with one retractable lever and a stimulation cable connected through a swivel commutator (Plastics One, Roanoke, VA, USA), and insulated wire to a constant current stimulator (Med Associates, Saint Albans, VT, USA). Each lever press resulted in a 500-ms train of 0.1-ms rectangular cathodal pulses through the electrode, followed by retraction of the lever and a 500 ms “time out” in which further presses did not produce brain stimulation. After acquiring brain stimulation-reinforced lever pressing, animals were presented with a series of 16 different pulse frequencies, ranging from 141 to 25 Hz in descending order. At each pulse frequency, animals could lever press for two 30-s trials, after which the pulse frequency was decreased by 0.05 log units. The response rate for each frequency was defined as the average number of lever presses during the two 30-s trials. The BSR threshold (*θ*_0_) was defined as the minimum frequency at which an animal responded for rewarding stimulation, calculated using the Gompertz sigmoidal model [5]. Y_max_ was defined as the maximal rate of responding (number of lever presses for BSR per unit of time).

*Effects of PIMSR and cocaine on electrical BSR*

After stable BSR responding was achieved (<10% variation in *θ*_0_ over 3 consecutive days), the testing phase began. Thirty min before the test session animals were injected systemically with one of the PIMSR doses (0, 3,10 or 30 mg/kg). During the test session animals could lever-press for brain stimulation in the presence or absence of cocaine (2 mg/kg, i.p.) treatment. After each test, animals received additional ICSS sessions until a new baseline *θ*_0_ was established. A different dose of PIMSR was then tested. The order of testing with different doses of PIMSR was counterbalanced.

**Exp. 5: Optogenetic intracranial self-stimulation (ICSS) in DAT-Cre mice**

To determine whether a dopamine (DA)-dependent mechanism underlies the effects of PIMSR on cocaine self-administration, we measured the effects of PIMSR on ICSS maintained by optical activation of ventral tegmental area (VTA) DA neurons in DAT-Cre mice expressing Cre-recombinase under the DA transporter (DAT) promoter.

***Surgery***

Surgery for intracranial optical fiber implantation for oICSS was the same as reported previously [6]. Briefly, mice were anesthetized with ketamine (90 mg/kg, intraperitoneal, i.p.) and xylazine (10 mg/kg, i.p.) and placed in a stereotaxic frame (David Kopf Instruments, Tujunga, CA, USA). For intra-VTA microinjection of virus, a custom-made 30-gauge stainless steel injector was used to infuse Cre-inducible recombinant AAV that encoded channel-rhodopsin (ChR2) and enhanced yellow fluorescent protein (AAV- EF1α-DIO-ChR2-EYFP; 150 nl, ∼2 × 10^12^ genomes/ml, University of North Carolina School of Medicine Gene Therapy Center, Chapel Hill, NC, USA) unilaterally into the VTA (AP −3.28; ML 0.43; DV −4.41 mm relative to Bregma) using a micropump (WPI 2000 UltraMicroPump, Sarasota, FL, USA) with a speed of 50 nl/min. A custom-built optrode (200-μm multimode optical fiber, Thorlabs, Newton, NJ, USA) tethered to an intracranial ceramic ferrule (MM-FER2007C-2300, Precision Fiber Products, Inc., Milpitas, CA, USA) was implanted into the VTA approximately 1 mm above the AAV injection site. To fix the optrode assembly to the skull, two screws were placed toward the front of the skull followed by a small layer of superglue. While the glue was still wet, dental cement was used to fix and seal the assembly together. Once the AAV vector injection and optrode implantation were finished, mice were given at least 4 weeks to recover from the surgery and to enable full AAV expression and ChR2 trafficking before oICSS experiments began.

***Procedures***

Mouse optical ICSS chambers (Med-Associates, Saint Albans, VT, USA) were equipped with two retractable levers, a light above the lever, two sheathed optic fibers (200-µm core diameter, Precision Fiber Products, Inc., Milpitas, CA, USA) connecting each mouse to a 473-nm wavelength laser (OEM Laser Systems, Midvale, UT, USA) by an FC/FC fiber rotary joint (Doric Lenses, Inc., Quebec, QC, Canada).

DAT-Cre mice with ChR2 expression in the VTA DA neurons were connected to a 473-nm wavelength laser (OEM Laser Systems, Midvale, UT, USA) by two sheathed optic fibers (200-µm core diameter, Precision Fiber Products, Inc., Milpitas, CA, USA) and FC/FC fiber rotary joint (Doric Lenses, Inc., Quebec, QC, Canada). The total output of the laser was adjusted to ∼10 mW transmittance into the brain. During each 1 h session, a press on the active lever activated the light above the lever and delivered a 50 Hz train of 5 ms-pulses of blue light to depolarize VTA DA neurons. Pressing the inactive lever had no consequence. When responding stabilized (<20% variability in responding for at least 3 consecutive sessions), a multiple stimulation frequency schedule was introduced. Every 10 min, the stimulation frequency was decreased from 100 to 50, 25, 10, 5 and finally to 1 Hz and lever presses at each frequency were counted.

*Effects of PIMSR on DA-dependent optical ICSS*

After consistent oICSS responding was reached on the multiple stimulation frequency schedule (with < 20% variation across 3 consecutive sessions), the testing phase began.  Mice received a systemic injection of PIMSR (0, 3, 10 or 30 mg/kg) 30 min prior to the test session and were allowed to lever-press for oICSS. After each test, mice received 3 additional oICSS sessions or until a new baseline was established and were re-tested with a different dose of PIMSR. The order of dose treatment was counterbalanced.

*Effects of PIMSR on Δ^9^-THC-induced inhibition of optical ICSS*

To further evaluate whether a CB1R-dependent mechanism underlies PIMSR action on oICSS, we examined the effects of PIMSR pretreatment on Δ^9^-THC-induced changes in oICSS in DAT-Cre mice. Briefly, a separate group of DAT-Cre mice were trained to lever-press for oICSS maintained by multiple stimulation frequencies. After a stable baseline was established, the ability of Δ^9^-THC to attenuate oICSS responding was evaluated at 1, 2 and 3.5 mg/kg i.p. Next, DAT-Cre mice were pretreated with 0, 10 or 30 mg/kg of PIMSR (i.p) 30 min before being injected with 3.5 mg/kg Δ^9^-THC. Fifteen min later their responding for oICSS was measured under the descending stimulation frequency schedule.

*Effects of PIMSR on ACEA-induced inhibition of optical ICSS*

Given that Δ^9^-THC is a non-selective CB1R agonist, we further examined the effects of PIMSR on ACEA (a selective CB1R agonist)-induced effects on oICSS in an additional group of DAT-Cre mice. We have previously reported that ACEA, at 1 or 3 mg/kg (i.p.), produced a significant reduction in oICSS in DAT-cre mice in a dose-dependent manor [6]. Therefore, in the present study, DAT-Cre mice were pretreated with vehicle, 3, 10 or 30 mg/kg of PIMSR (i.p) 30 min before being injected with 3 mg/kg ACEA after a stable baseline was established. Fifteen min later their responding for oICSS was measured under the descending stimulation frequency schedule.

*TH-immunostaining assays*

To determine whether AAV-ChR2-eYFP is expressed in VTA DA neurons, we used IHC to examine fluorescent eYFP co-localization with tyrosine hydroxylase (TH) in the midbrain of DAT-Cre mice. The IHC procedures were performed as reported previously [6]. Briefly, mice were deeply anesthetized with 100 mg/kg pentobarbital and transcardially perfused with cold saline followed by 4% paraformaldehyde in 0.1-M phosphate buffer. Brain tissue was transferred to 20% sucrose in phosphate buffer at 4°C overnight. Coronal sections were cut at 25 μm on a cryostat (CM3050S, Leica Microsystems Nussloch GmbH, Nussloch, Germany). Tissue sections containing the VTA were blocked and floated in 5% bovine serum albumin and 0.5% Triton X-100 phosphate buffer for 2 h at room temperature. Dual-labeling IHC was performed using an anti-TH monoclonal antibody (1:500; Millipore, Billerica, MA, USA). Sections were washed and incubated with a secondary antibody – goat anti-mouse Alexa 568 for TH (Millipore, #MAB318; 1:500) in 5% bovine serum albumin and 0.5% Triton X-100 phosphate buffer for 2 h at room temperature. Sections were washed, mounted, and cover slipped. Fluorescent images were captured using a fluorescence microscope (Nikon Eclipse 80i) equipped with a digital camera (Nikon Instruments Inc., Melville, NY, USA). All images were captured under identical optical conditions.

**Exp. 6: Δ^9^-THC-induced tetrad effects in mice**

Given the unexpected negative finding that PIMSR pretreatment failed to block Δ^9^-THC’s reward-attenuating effects in oICSS, we sought to determine whether PIMSR would antagonize Δ^9^-THC-induced cannabimimetic activity using the tetrad test [7].

*Analgesia:* Analgesia, defined as a decrease in thermal pain sensitivity, was assessed using a hot-plate device (Model 39, IITC Life Science Inc., Woodland Hills, CA, USA). A mouse was placed inside a transparent cage on the hot plate (55 ± 0.2 °C). The latency for the first sign of thermal nociception (licking, stomping the hind paw or jumping) was measured in seconds. The cut-off time for the test was 60 s to avoid tissue damage.

*Hypothermia:* Hypothermia or a decrease in rectal temperature (°C) was measured with a thermometer connected to a lubricated RET-2 rectal probe (Harvard Apparatus, Holliston, MA, USA) that was inserted into the rectum to a depth of 2-3 cm.

*Catalepsy:* Catalepsy, defined as an impaired capacity to initiate movements, was measured using an elevated bar test. Mice were suspended by their front paws from a metal bar (12 cm length) that was fixed horizontally at a height allowing their hind paws to just touch the floor. The latency to descend from the bar (i.e., when the two forepaws touched the floor) or when 3 min elapsed (i.e., cut-off time) was measured.

*Immobility:* Immobility was assessed using a rotarod device (Harvard Apparatus, Holliston, MA, USA) and measured as the latency to fall from the rotating platform under increasing speeds from 4 to 40 rev/min over 5 min.

*The effects of PIMSR on Δ^9^-THC-induced tetrad effects*

Baseline values for rectal temperature, hot-plate analgesia, catalepsy and rotarod motor coordination were determined.  Wild type mice (n=16) were divided into two groups – one group received Vehicle + Δ^9^-THC and another received 10 mg/kg PIMSR + Δ^9^-THC. Each animal received three doses of Δ^9^-THC (0, 10 or 30 mg/kg) in a randomized order with three washout days between doses. Mice were tested for hypothermia, analgesia, catalepsy and rotarod performance at 0 (baseline), 0.5, 1, 2 and 3 hours following Δ^9^-THC treatment. The sequence of tests was counterbalanced across the different timepoints.

**Data analysis**

All values were presented as means ± *SEM.* Animal group sizes were chosen based on a power analysis (*n* = 6-12 per group) and extensive previous experience with the animal models used. No data points were excluded from the analysis in any experiment. The investigators were blinded to the group allocation during the experiments and when assessing the outcome. To validate the use of parametric statistics, we ensured that our data were normally distributed and met variance homogeneity criteria with group sizes *n* > 5. Statistical analysis was done using the independent values coming from individual animals in each group. The group variances were similar between groups that were statistically compared. One-way repeated measures analysis of variance (RM ANOVA) was used to evaluate the effects of PIMSR on cocaine intake, cue-induced reinstatement of drug seeking and drug-induced changes in electrical or optical ICSS (AUC data). Two-way RM ANOVA was used to evaluate the effects of PIMSR on cocaine self-administration, Δ^9^-THC or ACEA-induced changes in oICSS, CPP, or locomotion. Three-way ANOVAs were used to analyze the drug effects over time, Δ^9^-THC dose, and group (Vehicle + Δ^9^-THC *vs*. PIMSR + Δ^9^-THC) in each tetrad measure. Significant Dose × Group × Time interactions were followed by Dose × Time interaction comparisons for each genotype. The post hoc group comparisons were conducted only if the ANOVA F value achieved *p*<0.05 and there was no significant variance in homogeneity. We also used the area under the curve (ΔAUC) to evaluate the tetrad effects of Δ^9^-THC in the absence or presence of PIMSR. Specifically, ΔAUC was measured as the sum of differences between each time point after the drug injection and the baseline before the injection. Group differences were analyzed using separate two-way (Group × Dose) ANOVAs, followed by tests of simple effect of PIMSR pretreatment for each Δ^9^-THC dose using one-way ANOVAs. The value of *p<*0.05 was used to indicate statistically significant differences among or between groups.





**Figure S1**: The effect of systemic administration of PIMSR on high-dose Δ^9^-THC-induced tetrad effects in wildtype mice. **A/B**: Time courses and ΔAUC data of Δ^9^-THC-induced hypothermia (as assessed by rectal temperature) (n = 8). A three-way RM ANOVA with groups (Δ^9^-THC *vs*. PIMSR + Δ^9^-THC) as a between-subjects factor and time and dose as within-subjects factors revealed a significant three way interaction (F_8,112_=2.35, *p*=0.02), which was driven by a significant effect of Δ^9^-THC alone across time (F_8,56_=10.59, *p*<0.001) and dose ( F_8,112_=17.5, *p*<0.001), suggesting that Δ^9^-THC significantly reduced body temperature over time, regardless of whether it was administered alone or with PIMSR (A). A two-way RM ANOVA over Δ^9^-THC dose for the ΔAUC data (B) revealed a main effect of Δ^9^-THC dose (F_2, 28_ = 3.87, *p*<0.05), but not PIMSR treatment (F_1, 14_ = 0.133, *p*>0.05) and no interaction between Δ^9^-THC dose × PIMSR treatment (F_2, 28_ = 0.43, *p*>0.05). **C/D**: Time courses and ΔAUC data of Δ^9^-THC-induced analgesia (as assessed by hot-plate testing) (n = 8). Similar data analyses (for the data shown in (**C**) revealed a significant time × dose interaction (F_8,112_=3.98, *p*<0.001), but no dose × group interaction (F_2,28_=0.86, *p*=0.43) or three-way time × dose × group interaction (F_8,112_=1.46, *p*=0.18) suggesting that Δ^9^-THC produced significant analgesic effects that PIMSR failed to block. The two-way RM ANOVA on Δ^9^-THC dose for the ΔAUC data (**D**) revealed a significant Δ^9^-THC dose main effect (F_2, 28_ = 22.67, *p*<0.001), but no significant PIMSR treatment main effect (F_1, 14_ = 0.033, *p*>0.05), nor THC dose × PIMSR interaction (F_2, 28_ = 0.041, *p*>0.05). **E/F**: Time courses and ΔAUC data for Δ^9^-THC-induced catalepsy (as assessed by the elevated bar test) (n = 8). A three-way ANOVA for the catalepsy data (**E**) revealed a significant time × dose interaction (F_8,112_=11.11, *p*<0.001), but no dose × group (F_2,28_=0.35, *p*=0.70), or three-way interaction (F_8,112_=0.65, *p*=0.73). This indicates that Δ^9^-THC produced significant catalepsy, regardless of whether it was administered alone or with PIMSR. A two-way RM ANOVA for the ΔAUC data (**F**) revealed a Δ^9^-THC dose main effect (F_2, 28_ = 9.94, *p*<0.001), but no PIMSR treatment main effect (F_1, 14_ = 0.062, *p*>0.05) or Δ^9^-THC dose × PIMSR interaction (F_2, 28_ = 0.499, *p*>0.05). **G/H**: Time courses and ΔAUC data of Δ^9^-THC-induced immobility (as assessed by rotarod performance) (n = 8). A three-way ANOVA on the rotarod data (**G**) revealed a significant time × dose interaction (F_8,112_=15.22, *p*<.001), but no time × dose × group (F_8,112_=1.3, *p*=0.25) or dose × group interactions (F_2,28_=1.48; *p*=0.24). Δ^9^-THC significantly impaired the rotarod performance in the presence or absence of PIMSR. A two-way RM ANOVA on the ΔAUC data (**H**) showed a Δ^9^-THC dose main effect (F_2, 28_ = 101.18, *p*<0.001), but no PIMSR treatment main effect (F_1, 14_ = 1.34, *p*>0.05) or THC dose × PIMSR interaction (F_2, 28_ = 0.36, *p*>0.05). ****p*<0.01, as compared to the vehicle control group **(A, C, E, G**) or 0 mg/kg Δ^9^-THC (**B, D, F, H**).

Overall, PIMSR, at 10 mg/kg, failed to alter high dose Δ^9^-THC-induced tetrad effects (analgesia, hypothermia, catalepsy and immobility).

**References:**

1. Richardson NR, Roberts DCS. Progressive ratio schedules in drug self-administration studies in rats: a method to evaluate reinforcing efficacy. Journal of Neuroscience Methods. 1996; 66:1–11.
2. Xi Z-X, Spiller K, Pak AC, Gilbert J, Dillon C, Li X, et al. Cannabinoid CB1 receptor antagonists attenuate cocaine’s rewarding effects: experiments with self-administration and brain-stimulation reward in rats. Neuropsychopharmacology. 2008; 33:1735–1745.
3. Bi GH, Galaj E, He Y, Xi ZX. [Cannabidiol inhibits sucrose self-administration by CB1 and CB2 receptor mechanisms in rodents.](https://pubmed.ncbi.nlm.nih.gov/31215752/) Addict Biol. 2020; 25:e12783.
4. Spiller K, Bi GH, Galaj E, Gardner EL, Xi ZX. Cannabinoid CB1 and CB2 receptor mechanisms underlie cannabis reward and aversion in rats. Br J Pharmacol. 2019. 2019.
5. Coulombe D, Miliaressis E. Fitting intracranial self-stimulation data with growth models. Behav Neurosci. 1987; 101:209–214.
6. Humburg BA, Jordan CJ, Zhang H-Y, Shen H, Han X, Bi G-H, et al. Optogenetic brain-stimulation reward: A new procedure to re-evaluate the rewarding versus aversive effects of cannabinoids in dopamine transporter-Cre mice. Addict Biol. 2021; e13005.
7. Wang XF, Galaj E, Bi GH, Zhang C, He Y, Zhan J, et al. [Different receptor mechanisms underlying phytocannabinoid- versus synthetic cannabinoid-induced tetrad effects: Opposite roles of CB_1_ /CB_2_ versus GPR55 receptors.](https://pubmed.ncbi.nlm.nih.gov/31877572/) Br J Pharmacol. 2020; 177:1865-1880.
